# Use of COVID-19 Convalescent Plasma for Treatment of Symptomatic SARS-CoV-2 Infection at a Children’s Hospital: A Contribution to a Still Inadequate Body of Evidence

**DOI:** 10.3390/children10020350

**Published:** 2023-02-10

**Authors:** Antonio Arrieta, Alvaro E. Galvis, Stephanie Osborne, Tricia Morphew, Karen Imfeld, Claudia Enriquez, Janet Hoang, Marcia Swearingen, Delma J. Nieves, Negar Ashouri, Jasjit Singh, Diane Nugent

**Affiliations:** 1 Pediatrics Infectious Diseases, CHOC Children’s Hospital, Orange, CA 92868, USA; 2 Department of Pediatrics, University of California Irvine School of Medicine, Irvine, CA 92697, USA; 3 Research Administration, CHOC Children’s Hospital, Orange, CA 92868, USA; 4 Morphew Consulting, LLC, CHOC Research Institute, CHOC Children’s Hospital, Orange, CA 92868, USA; 5 Hematology Advanced Diagnostics Laboratory, CHOC Children’s Hospital, Orange, CA 92868, USA; 6 Pediatric Hematology, CHOC Children’s Hospital, Orange, CA 92868, USA

**Keywords:** convalescent plasma, COVID-19 treatment, symptomatic SARS-CoV-2, pediatrics

## Abstract

Data on COVID-19 convalescent plasma (CCP) safety and efficacy in children and young adults are limited. This single-center prospective, open-label trial evaluates CCP safety, neutralizing antibody kinetics, and outcomes in children and young adults with moderate/severe COVID-19 (April 2020–March 2021). A total of 46 subjects received CCP; 43 were included in the safety analysis (SAS); 7.0% < 2 years old, 2.3% 2–<6, 27.9% 6–<12, 39.5% 12–<19, and 23.3% > 19 years old; 28 were included in the antibody kinetic analysis (AbKS); 10.7% < 2 years old, 10.7% 6–<12, 53.8% 12–<19, and 25.0% > 19 years old. No adverse events occurred. The median COVID-19 severity score improved (5.0 pre-CCP to 1.0 by day 7; *p* < 0.001). A rapid increase in the median percentage of inhibition was observed in AbKS (22.5% (13.0%, 41.5%) pre-infusion to 52% (23.7%, 72%) 24 h post-infusion); a similar increase was observed in nine immune-competent subjects (28% (23%, 35%) to 63% (53%, 72%)). The inhibition percentage increased until day 7 and persisted at 21 and 90 days. CCP is well tolerated in children and young adults, providing rapid and robust increased antibodies. CCP should remain a therapeutic option for this population for whom vaccines are not fully available and given that the safety and efficacy of existing monoclonal antibodies and antiviral agents have not been established.

## 1. Introduction

COVID-19, the clinical syndrome associated with SARS-CoV-2 infection, has disproportionately affected adults yet, although less likely to be symptomatic, an estimated 85,000 children have died [1]. Several risk factors for severe disease among children, adolescents, and young adults (child/AYA) have been identified. No treatment options were available early in the pandemic, although eventually remdesivir and later low-dose steroids improved survival in adults [2,3]. None of these regimens have been formally studied in pediatrics. The use of COVID-19 convalescent plasma (CCP) was adopted early; however, multiple publications have presented diverse and often contradictory results, although it is broadly accepted that CCP is safe in adults [4,5,6,7].

Faced with limited options, in April 2020 we obtained emergency use authorization (EUA) from the Food and Drug Administration (FDA) and developed a CCP treatment protocol. We have previously reported on the safety and anti-nucleocapsid antibody kinetics of CCP in a small cohort of pediatric (<18 years old) patients [8]. Here we present the impact of passive neutralizing antibody (Nab) infusion in a larger cohort of child/AYA (2 months–22 years old) hospitalized with COVID-19. 

## 2. Materials and Methods

### 2.1. Study Design

A prospective, open-label treatment trial was conducted at the Children’s Hospital of Orange County (CHOC), Orange, California. Approval under eINDs was obtained from the FDA and the local institutional review board. Hospitalized patients < 26 years old, with COVID-19 confirmed by positive SARS-CoV-2 polymerase chain reaction (PCR) from nasopharyngeal (NP) swab were eligible to receive CCP, 10 mL/kg up to 1 unit (approximately 260 mL) if they met any of the following criteria: (1) hospitalized requiring oxygen; (2) oxygen saturation < 93% on ambient air; (3) partial pressure aO2:FIO2 ratio < 300; and/or (4) pulmonary infiltrates > 50% within 24–48 h of admission. Signed informed consent/assent was obtained from participants in the antibody kinetics study; receiving CCP otherwise required only informational documentation.

### 2.2. Serum/Plasma Samples

Samples were obtained from CCP at the completion of infusion and from patients pre-CCP, 24 h, 7 days, 21 days, and 3 months post-infusion. Samples were stored at 4 °C and analyzed within 8 days, after which samples were stored at −20 °C with only a single freeze–thaw cycle.

#### CCP Donors

Per American Red Cross guidelines, donors were eligible to provide CCP if: (a) they were initially proven positive for SARS-CoV-2 by a laboratory test; and were either (b1) > 14 days from symptom resolution with repeat documented negative test for SARS-CoV-2, or (b2) > 28 days from symptom resolution without repeat test results at the time of plasma collection. CCP was collected and stored at the CHOC blood bank.

### 2.3. Study Population

Demographic data and co-morbidities were abstracted from the hospital’s electronic medical record. Comorbidities were identified by documented primary diagnosis with associated ICD10 codes and/or health encounters at related clinics or by the record of visits to specialty care clinics for the treatment of asthma, diabetes, neurologic, oncologic, cardiac, or hematologic conditions; obesity was defined as BMI > 95th percentile for age/gender [9].

### 2.4. SARS-CoV-2 Surrogate Virus Neutralization Test (sVNT)

The SARS-CoV-2 sVNT from GenScript (Piscataway, NJ, USA, catalog #L00847-A) was used to detect NAb targeting the viral spike (S) protein receptor binding domain. The test is based on antibody-mediated blockage of the interaction between the angiotensin-converting enzyme 2 (ACE2) receptor protein and the receptor binding domain. The test was performed according to the manufacturer’s instructions. Samples and controls were tested in duplicate. Cutoff values were determined according to the manufacturer’s instructions. Units per milliliter (U/mL) were calculated using the SARS-CoV-2 Neutralizing Antibody Standard from GenScript (catalog #A02087-100) with the sVNT kit. The antibody standard was tested at a 600 U/mL starting concentration with six additional twofold serial dilutions. Unknowns were calculated from the curve using GraphPad Prism (San Diego, CA, USA). The inhibition percentage (%-inhibition) was calculated as [1 −  (OD value of sample/OD value of Negative control)]  ×  100.

### 2.5. Outcomes Measurement

The primary outcome measured was to assess the safety of CCP administration for the treatment of severe to critically ill hospitalized pediatric, adolescent, and young adult patients with COVID-19. All patients who completed CCP infusion and had safety data collected are included in the safety analysis set (SAS). Vital signs and oxygen requirements were monitored during the infusion and over the following 24 h to capture infusion-related adverse events (AEs) such as transfusion-related acute lung injury (TRALI), transfusion-associated circulatory overload (TACO), antibody-dependent enhancement of disease (ADE), or allergic reactions. TRALI was impossible to evaluate as all patients had acute lung injury (ALI) before CCP infusion, so worsening of ALI was captured instead (see Supplementary Table S1 for definition criteria).

Safety laboratory tests (white blood cell (WBC) count, hemoglobin (Hgb), platelets count, sodium (Na), potassium (K), creatinine, aspartate aminotransferase (AST), alanine aminotransferase (ALT), and bilirubin) were obtained before infusion, 24 h, and 7 days (or on day of discharge if before 7 days) after infusion from patients consented to participation in the antibody kinetics dataset (AbKS). 

Secondary outcomes measured included NAb %-inhibition kinetics. Though not intended as an efficacy evaluation of CCP, clinical response to treatment is reported. All patients who signed consent for inclusion and had kinetic and safety laboratory tests obtained per protocol were included in the AbKS. The median percentage of inhibition from CCP and patients at the intervals noted above were compared to describe Nab kinetics following infusion. Response to treatment was measured by WHO severity scores before infusion and on day 7 or at discharge (if prior to day 7). The scoring as previously reported is as follows: 1—not hospitalized, no limitations on activity; 2—not hospitalized, limitations of activity (e.g., home O_2_ requirement); 3—hospitalized, no O_2_ and/or no longer requiring medical care; 4—hospitalized, no O_2_, but requiring medical care (COVID-19 related or not); 5—hospitalized, requiring O_2_; 6—hospitalized, requiring NIV or use of high flow; 7—hospitalized, requiring invasive mechanical ventilation or ECMO; and 8—hospitalized, expired before discharge [2]. In addition to safety laboratory tests, C-reactive protein (CRP), ferritin, and D-dimers were obtained as clinically indicated.

### 2.6. Statistical Analysis

Descriptive results were expressed as numbers and percentages with a defined trait or mean (SD) or median (IQR) for continuous measures, as appropriate. Friedman’s test assessed the significance of the reduction in median COVID-19 severity from admission to pre-CCP, 3 days, and 7 days post-CPP. The distribution of donor CCP percentage inhibition was plotted and compared to plasma-blood CCP and post-transfusion follow-up time points of 24 h, 7 days, 21 days, and 3 months. The time at which the difference in average percentage of inhibition became significant in relation to donor CCP level was based on GEE analyses with the specification of gamma distribution due to positive skewness of data, repeat measures, and independent correlation structure. Analyses were performed using SPSS v27.0 [10]. 

## 3. Results

From 1 April 2020 to 31 March 2021, 530 patients were admitted to CHOC with positive NP-PCR for SARS-CoV-2, and 93 (17.5%) were diagnosed with COVID-19. A total of 46 patients received CCP, 30 were approached for participation in AbKS, and 3 patients were later excluded (one died, one due to parental request, and one with Bruton’s agammaglobulinemia). Among these, 43 were included in SAS and 28 in AbKS (4 patients with BMI > 35 who received 2 CCP infusions were excluded from the AbKS and are reported separately).

In the SAS, 24 (55.8%) patients were male, 34 (79.1%) Hispanic; 3 (7.0%) were <2 years old, 1 (2.3%) was 2–6 years old, 12 (27.9%) were 6–12 years old, 17 (39.5%) were 12–18 years old, and 10 (23.3%) were 19–22 years old (median, Q1, Q3 = 14, 8.5, 18). A total of 39 patients received 1 infusion (26 received 1 unit, and 13 were planned to receive 10 mL/kg, see below). Four patients (body weight = 120 kg, 158.3 kg, 146.4 kg, and 92.7 kg) received two one-unit infusions. Due to variations in the volume of CCP collected from each donor, there was variability in total and per kilo volume infused per patient. Mean (SD) volume in mL/Kg received by patients < 25 kg (expected to receive 10 mL/kg) was 8.7 (2.3) mL/kg, while those > 25 kg (1 unit) (mean weight 88.9 (31.6) kg) received a mean (SD) volume of 2.9 (1.1) mL/kg. During this period, our institution had no ability to measure SARS-CoV-2 NAb titers in CCP prior to the release of the product from the blood bank. The %-inhibition in CCP reported in these datasets was analyzed retrospectively. Overall co-morbidities were present in 41 (95.4%) patients (Table 1).

No patient experienced infusion-related AEs or allergic reactions. No patient had clinical evidence of ADE. Safety laboratory tests selected did not exhibit clinically significant change at 24 h, 7, and 21 days after infusion (Table 2).

In the AbKS, 15 (53.6%) were male; 25 (89.3%) were Hispanic; 3 (10.7%) patients were <2 years old, none were 2–6 years old, 3 (10.7%) were 6–12 years old, 15 (53.8%) were 12–19 years old, and 7 (25.0%) were 19–22 years old. Four patients received two infusions, and their antibody kinetics are presented separately. Four subjects were immune-compromised (two bone marrow transplants, one with acute lymphocytic leukemia (ALL), and one with ALL and cystic fibrosis). Morbid obesity (BMI > 35 kg/m^2^ or >99th percentile for age) was a common comorbidity present in 12 (42.9%) patients.

**Figure 1:**
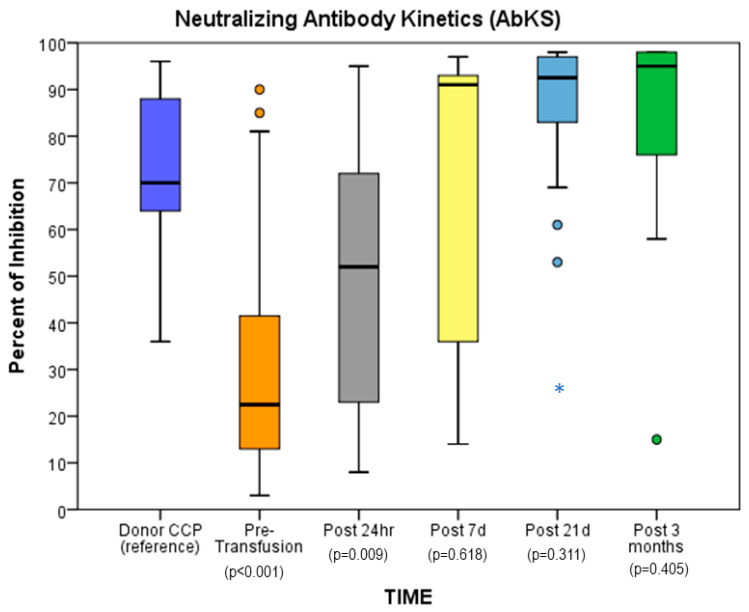
Average percentage of inhibition in relation to donor CCP depended on time since infusion (*p* < 0.001), N = 24. Significance based on GEE analyses with the specification of gamma distribution, repeat measures, and independent correlation structure. The dots represent outliers. The asterisk represents an extreme outlier.

The 24 patients with a single CCP infusion had significantly lower pre-infusion average %-inhibition compared to CCP through post-24 h (*p* < 0.05); the average value increased to similar %-inhibition to CCP by 7 days and was maintained through 3 months (*p* ≥ 0.05). (Figure 1)

Median (IQR: Q1, Q3) %-inhibition in CCP was 70.0% (IQR: 64.0%, 88.0%); CCP recipients’ median %-inhibition pre-CCP infusion was 22.5% (IQR: 13.0, 41.5). At 24 h following CCP infusion, this value was 52.0% (IQR: 23.7%, 72.0%), significantly higher than baseline; %-inhibition on days 7 and 21 post CCP infusion was 91.0% (IQR: 36.0%, 93.0%) and 92.5% (IQR: 83.0%, 97.0%), respectively. At 3 months, the median %-inhibition remained high at 95.0% (IQR: 76.0%, 98.0%). After two CCP infusions, a similar increase in %-inhibition was observed in four patients with morbid obesity, with a difference of 16.4 percentage points in the median %-inhibition of the two post-24 (Table 3). Safety in this small cohort was not different than observed in the full SAS cohort.

**Figure 2:**
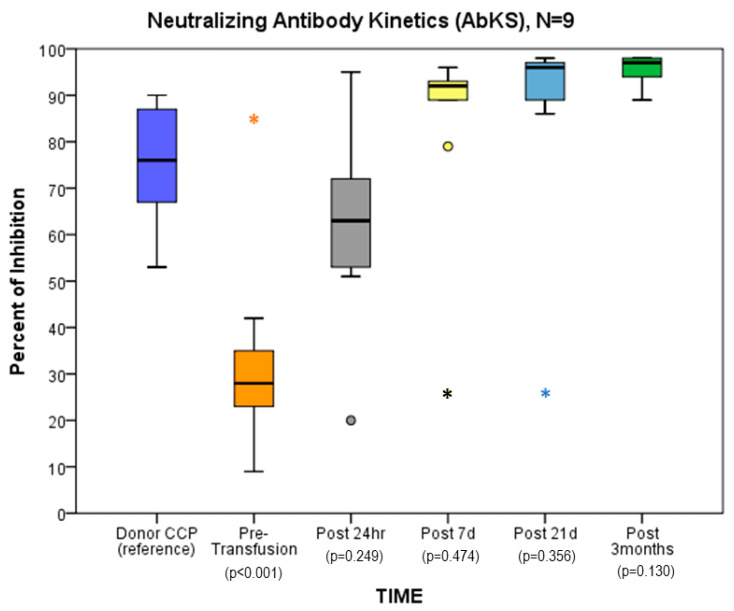
In 9 immune-competent hosts without underlying disease and a full dataset to three months out, average percentage of inhibition in relation to donor CCP depended on time since infusion (*p* < 0.001). Significance based on GEE analyses with the specification of gamma distribution, repeat measures, and independent correlation structure. The dots represent outliers. The asterisk represents an extreme outlier.

A group of nine immune-competent hosts without underlying disease and a full dataset three months out showed a rapid and statistically significant increase in %-inhibition starting 24 h after CCP, similar to the full cohort, which continued to day 7 and persisted at 21 days and 3 months (Figure 2).

**Figure 3:**
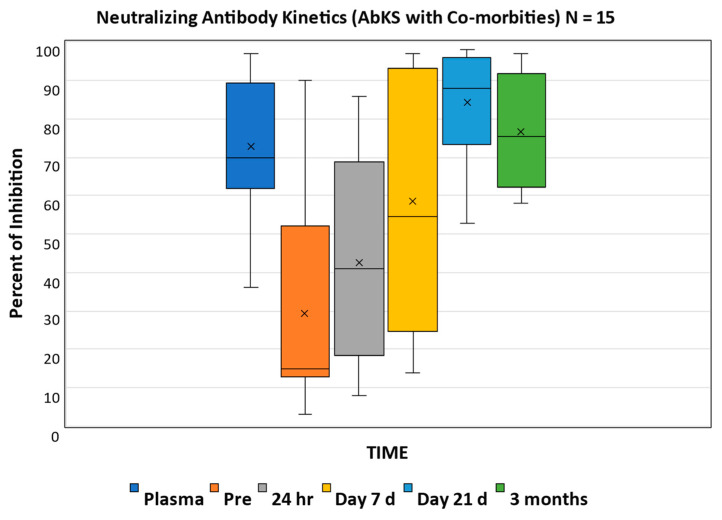
Percentage of inhibition over time in hosts with co-morbidities shows a substantial increase in median value at 24 h post-infusion (15% to 41%); the change from 24 h to 7 days (41–54.5%) was modest and below the median % inhibition of CCP. Only at day 21, a substantial increment is noted compared to 24 h after CCP (41–88%). The mean is identified by “x”.

Fifteen patients with co-morbidities that included immune-suppressive conditions showed a similar increase in median %-inhibition after CCP infusion (15–41%) immediately after CCP infusion, but the increase from 24 h to 7 days was modest (41–54.5%) compared to the whole cohort (51–91%) and to the cohort of immune-competent patients (63–92%) (Figure 3).

**Figure 4:**
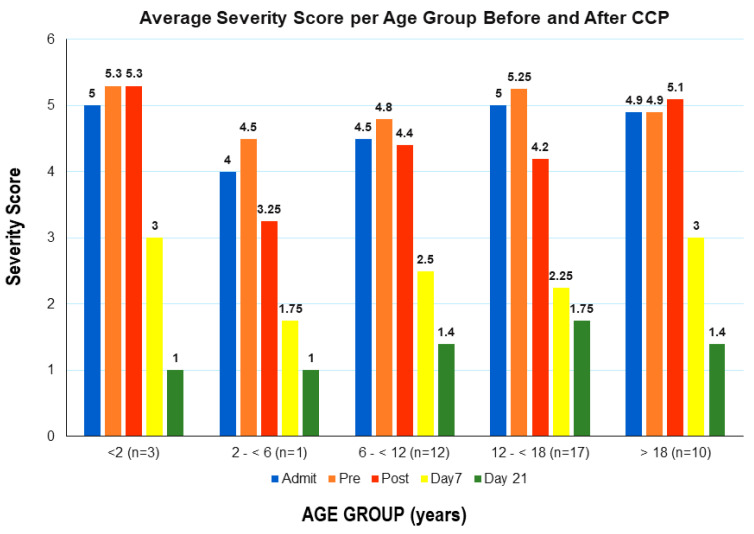
Severity score for all age groups remained similar or declined at 24 h post-CCP infusion supporting the safety of CCP. By day 7 score had substantially improved (3—no oxygen). Most patients had been discharged to home (1—home) by day 21. The numbers in each group were too small to allow for statistical analysis.

The median hospital length of stay was 7.0 days (IQR: 5.5, 10.0) and the ICU length of stay (16/28 patients required ICU) was 5.0 days (IQR: 2.0, 7.5). Pre-CCP median COVID-19 severity score was 5.0 (IQR: 5.0, 6.0), which reduced slightly to 4.5 by day 3 (IQR: 3.5, 5.0), *p* = 0.248, and decreased significantly to 1.0 by day 7 or at discharge (IQR: 1.0, 4.0), *p*< 0.001. The favorable change in severity score was similar for all age groups (Figure 4).

## 4. Discussion

SARS-CoV-2 infection in child/AYA is frequently asymptomatic or mild, though hospitalization and deaths have occurred [1,11,12,13,14]. Infants and adolescents are at higher risk of poor outcomes including death, particularly when risk factors (obesity, neuromuscular disease, and cardiac and chronic lung disease) are present [13,14,15]. Unlike for adults, data for children on antiviral and immune-modulating agents with regard to safety, efficacy, and pharmacokinetics are lacking, and access to preventive strategies such as monoclonal antibodies remains limited due to age (>12 years old) and/or weight (>40 kg) barriers. Even access to vaccines, now available to young children, is mainly limited to the US and Western Europe, where vaccine acceptance remains low.

Early in the pandemic, several antiviral treatments were tried with no clear therapeutic success yet significant risk for serious toxicities [16,17,18]. Later, remdesivir showed benefits in adults compared to the placebo [2]. Moreover, low-dose dexamethasone administered to adult patients who remained symptomatic after day 7 of illness, when the viral replication phase had subsided (>day 7), showed a significant survival advantage compared to placebo [3]. Similar studies evaluating the benefits of steroids were halted and analyzed early resulting in steroids becoming the standard of care [19,20,21]. Other immune-modulating treatment options have been evaluated (tocilizumab, JAK inhibitors) with mixed results [22,23,24]. Studies conducted with SARS-CoV-2 monoclonal antibodies highlight the efficacy of passive immunotherapy in the treatment of mild disease and in the prevention of disease progression from asymptomatic infection to a disease requiring admission to hospital. Their efficacy has been shown to be variant-specific and most have no benefit against Omicron and its subvariants. Access restriction to >12 years old and/or >40 kg, further limits therapeutic options for children [25,26,27]. Recent data on the transplacental transfer of anti-SARS-CoV-2 antibodies, from the mRNA vaccination of pregnant mothers to their newborns, showed protection from hospitalization and critical illness compared to infants born to mothers who were not immunized during pregnancy, further highlighting the protection conferred by passive immunity. Protection was noted for Delta and Omicron variants [28]. 

Several large (up to >25,000 patients) reports on the safety of CCP in adults are available but its efficacy has remained controversial [6]. Broad consensus exists that if used early in the course of illness (<4 days from illness onset), high NAb titer CCP will reduce mortality and length of hospitalization [29]. Variability in the timing of administration, the dose administered (one unit), and neutralizing antibody content of CCP in adult studies complicate the generalization of findings [5].

These studies indicate that underdosing of CCP is a common problem, since a discrepancy between the variant that infected the donor and the one infecting the recipient affects efficacy. They also show that passive immune therapy works only if administered before the recipient produces their own NAb, hence the need for administration within 4 days of onset of illness. A study in macaques showed that low doses (25 mg/kg) and high doses (250 mg/kg) of CCP containing 1/1581 NAb titer prevented symptomatic infection, but only the higher dose was efficacious in treating the disease. Extrapolation from the macaques’ data suggests that a patient weighing 70 kg would require >1300 mL of CCP with a NAb titer of 1/1280 to reach the NAb concentration provided to animals with the 250 mg/kg dose [30]. A study in hamsters showed that a high dose (40% of plasma volume) with human CCP 1/2560 NAb titer given 24 h after infection protected the animals from disease, suggesting that, for efficacy in humans, a CCP NAb titer of 1/1600 would be necessary for a 300 mL (approximately one unit of CCP) dose or 600 mL (two units) if the NAb titer is 1/800 [31].

Limited pediatric data on CCP exists, mainly focused on safety [8,32,33,34,35]. We recently reported our experience in a small cohort of children <18 years old. We showed that CCP in children is safe. Though larger than other pediatric studies, our sample size was still small, and the absence of controls limited our ability to discuss efficacy. Importantly, we reported that the patient’s ability to synthetize their own anti-nucleocapsid antibody was unaffected by CCP administration. Gordon et al. reported similar safety and antibody kinetics in 14 pediatric patients (9 treatment and 5 prophylaxis). In their report, a lower CCP dose (5 mg/kg) was used compared to ours, but they were able to ascertain a high NAb titer [35]. In that study, they were able to differentiate donor (CCP) from recipient antibodies and found that a fraction of donor antibodies was present in CCP recipients 3 h after infusion.

In this report, we expand our cohort to include older patients, specifically adolescents and young adults, who have been reported to have a higher risk of severe disease. We thought it was important to include this group as they are severely under-represented in adult studies. This time we measured anti-spike protein NAb by %-inhibition present in CCP and in patients before and after infusion (pre-infusion, 24 h, 7 days, 21 days, and 3 months after infusion). Our data show that early high NAb titer CCP infusion elicits passive NAb transfer to recipients. We show that early high NAb titer CCP infusion elicits fast passive immunity in patients who had low or no (<20% neutralization) NAb detected early in their illness. The presence of NAb has been associated with a decrease in SARS-CoV-2 viral load [4] and CCP provides a fast and robust increase in NAb immediately after infusion. The dose of CCP reported in other pediatric studies (4–5ml/kg) is lower than the 10 mL/kg we used. In adult studies, CCP infusion has been limited to one unit (approximately 300 mL) [4,36,37,38]. Most of our patients (>70%) were morbidly obese (BMI > 35 kg/m^2^). We became concerned that in these patients a one-unit dose would be insufficient. Upon review of the volume administered, we noticed that recipients > 25 kg who received one unit (dose used in adult studies) had received a relatively small volume of CCP (mean = 2.9 mL/kg); as discussed above, based on animal data, this would adversely impact the total amount of antibodies administered unless CCP contained high NAb titer. We administered two units of CCP 24 h apart to four morbidly obese patients (weight range 92.7–158.3 kg) and were able to identify an additive effect on the increase in %-inhibition in these patients. Safety remained excellent as no adverse events occurred. Concerns with safety in patients already exhibiting respiratory distress, sometimes with underlying cardiovascular disease, have guided the dose to be used in most adult studies, which may limit the amount of NAb administered. The safety of CCP has consistently been documented in all adult studies. We think the dose (volume in mL/kg) of CCP should be adjusted to the NAb titer present in CCP rather than the highly variable unit of CCP. 

We enrolled nine subjects who were immune-competent, received a single CCP infusion, had no underlying illness, and were able to provide specimens for all time points (full set). The rapid increase 24 h after infusion in %-inhibition was even more evident in this cohort who by day 7 had consistently developed a robust NAb response, which was sustained at 21 days and 3 months, indicating that the benefit of CCP is not limited to immune-compromised hosts as current guidelines suggest. Conversely, when we evaluated the antibody kinetics in patients with diverse co-morbidities which may impair antibody production (immune deficiencies, cancer, CLD, CKD, and congenital heart disease), we noticed a substantial increase in %-neutralization 24 h after infusion and only a modest increase in neutralizing antibodies by day 7.

We did not identify any clinically significant adverse events associated with CCP infusion, and, specifically, none of our patients showed signs of TRALI or TACO; no allergic reactions were noted including in those who received two infusions. Safety laboratory studies were not clinically different between baseline and day 21. Since we had no controls, we were not trying to assess efficacy, yet most patients experienced rapid clinical improvement; 14 (50%) of AbKS patients were discharged on ambient air in <7 days. Of 46 patients treated with CCP, there was one poor outcome—a 138 kg female patient with Prader–Willi (BMI 56) who arrived at our emergency room in cardiac arrest and was pronounced brain dead shortly after the CCP infusion.

Our data may be of greatest importance in developing nations, where immunization is lagging and antiviral agents, including monoclonal antibodies, are often unavailable or unaffordable. Antiviral therapeutic modalities have shown marginal benefit compared to placebo. The WHO does not endorse the use of remdesivir for the treatment of COVID-19 (it recently endorsed 3 days of remdesivir for the prevention of progression to serious illness) [5]. Furthermore, CCP likely has NAb against SARS-CoV-2 variants circulating in communities where CCP would be obtained from patients who recovered from recent infection.

Our study has significant limitations; importantly, the small sample size in comparison to adult studies. Another limitation is that we did not have a control group. In the absence of effective treatments when we started this treatment protocol and based on emerging adult data, we believed we had to provide all patients with safe and potentially effective treatment. The age range of our patients may be seen as a limitation, but patients 18–25 have either been excluded or represent a very small proportion in the adult studies. These adolescents and young adults exhibit different risk factors than younger children and older adults; particularly in our study, they were frequently morbidly obese, and need to be included in treatment trials.

## 5. Conclusions

In conclusion, we pediatricians had to face this pandemic with minimal data available and rapidly develop treatment strategies to manage patients with a strong emphasis on safety, as most children were expected to do well without treatment. There are still only minimal data on remdesivir, steroids, and other immune-modulatory agents for COVID-19 in children. Current pediatric CDC/NIH guidelines include a recommendation against the use of CCP for children hospitalized with COVID-19 except in clinical trials [39]. We believe that, in pediatrics, CCP remains a valid treatment option on a case-by-case basis, albeit one with a narrow therapeutic window, limited to situations when high NAb titer CCP can be provided early and appropriately dosed to patients with limited options. In the US, this applies to situations when due to age and weight, patients at risk for progression to severe disease still have limited access to evolving treatment modalities such as monoclonal antibodies or antiviral agents. It is likely that CCP will be the only antiviral option available to children in developing countries, likely to continue to experience large numbers of COVID-19 cases, particularly in pediatric patients who will be last to be immunized.

## Figures and Tables

**Table 1:** Demographic (N = 43).

**Age (Years)**	
median (IQR)	14 (8.18)
Range: N (%)	
<2	3 (7.0%)
2–6	1 (2.3%)
6–<12	12 (27.9%)
12–<18	17 (39.5%)
>18	10 (23%)
**Male** (N, %)	24 (55.8%)
**Hispanic**	34 (79%)
**Weight** (kg)	
Median (IQR)	70.3 (8.18)
Range	3.4–159
**BMI**	
Median (IQR)	27.35 (18.7, 38.1)
N (%) > 30	19 (45.2)
**Co-morbidities** * N (%)	41 (95.4)
Obesity	17 (39.6)
Oncology	7 (16.3)
Immune deficiency	5 (11.6)
Neuromuscular	6 (13.9)
CLD	5 (11.6)
CKD	1 (2.3)
Prematurity	2 (4.6)
Cardiac	2 (4.6)

* 5 (11.6%) patients had 2 comorbidities.

**Table 2:** Safety laboratory tests baseline to day 21 (mean + SD).

	Baseline	24 h	7 Days	21 Days
**WBC** (K/UL)	6.1 (4.0)	5.8 (4.3)	7.0 (4.2)	6.6 (4.2)
**Hgb** (gm/dL)	12.2 (2.3)	12.5 (3.4)	12.5 (3.4)	122.5 (3.4)
**Platelets** (K/UL)	173.2 (99.9)	206.9 (125.8)	294.3 (184.4)	247.3 (196.9)
**Creatine** (mg/dL)	0.8 (1.7)	0.8 (1.7)	0.5 (0.8)	0.5 (0.7)
**AST** (units/L)	63.7 (51.8)	52 (35.4)	54.9 (47.4)	68.6 (116.7)
**ALT** (units/L)	54.1 (69.4)	43 (49.1)	59.5 (65.5)	90 (137.4)

**Table 3:** Distribution of percentage inhibition across time in 4 patients who received two CCP infusions due to morbid obesity. Values listed are percentage inhibition.

	CCP 1	Pre	24 h Post 1	CCP 2	24 h Post 2	Post 7 d 2	Post d 21
Median	79	18.5	57.1	74.5	73.5	94.5	95.5
Q1	56.25	11	39.75	57.75	67.25	92.5	94.25
Q3	90.25	32	77.75	66.75	81.75	95.25	96.5

## Data Availability

Data available on request due to restrictions eg privacy or ethical. The data presented in this study are available on request from the corresponding author.

## Supplementary Materials

The following supporting information can be downloaded at: https://www.mdpi.com/article/10.3390/children10020350/s1. Table S1. Definitions of TRALI, TACO, allergic reactions and ADE. References [40,41,42,43,44] are cited in the supplementary materials.
