# Supplementary material for: Use of COVID-19 Convalescent Plasma for Treatment of Symptomatic SARS-CoV-2 Infection at a Children’s Hospital: A Contribution to a Still Inadequate Body of Evidence
Source: Children (Basel). 2023 Feb 10;10(2):350. doi: 10.3390/children10020350 (PMC9955755; doi:10.3390/children10020350)
Supplement: Supplementary file 1 [file children-10-00350-s001.zip › children-2104874-supplementary.pdf]

***Supplementary Table S1.* Definitions of TRALI, TACO, allergic reactions and ADE.**

**Transfusion Associated Lung Injury (TRALI)**[1-3]<br><br>NO evidence of worsening acute lung injury prior to transfusion<br><br>AND *Worsening* ALI onset during or within 6 hours of cessation of transfusion<br><br>AND *Worsening* hypoxemia (change in oxygen delivery/ventilatory support)<br><br>AND *Radiographic* evidence of bilateral infiltrates<br><br>AND no evidence of *worsening* left atrial hypertension
**Transfusion Associated Circulatory Overload (TACO)**[1, 2, 4]<br><br>New onset or exacerbation of 3 or more of the following within 12 hours of cessation of transfusion:<br><br>Evidence of acute worsening respiratory distress (dyspnea, tachypnea, cyanosis and decreased oxygen saturation values in the absence of other specific causes)<br><br>AND/OR radiographic or clinical evidence of acute or worsening pulmonary edema<br><br>AND elevated brain natriuretic peptide (BNP) or NT-pro BNP relevant biomarker<br><br>AND/OR other unexplained cardiovascular changes such as elevated central venous pressure
**Allergic Reaction**[2]<br><br>2 or more of the following occurring during or within 4 hours of cessation of transfusion (non-severe allergic reactions do not need to be reported):

Conjunctival edema

Edema of lips, tongue, and uvula

Erythema and edema of the periorbital area

Localized angioedema

Maculopapular rash

Pruritus (itching)

Urticaria (hives)

Antibody-Dependent Enhancement of Disease (ADE)[5]

Enhancement of disease severity in an infected person when an antibody against a pathogen—whether acquired by an earlier infection, vaccination or passive transfer—worsens its virulence by a mechanism that is shown to be antibody-dependent.

References:

1. Semple JW RJ, Kapur R. Transfusion-associated circulatory overload and transfusion-related acute lung injury. Blood. 2019(Apr 25;133(17):1840-1853).
2. CDC. National Healthcare Safety Network Biovigilance Component Hemovigilance Module Surveillance Protocol. 2021.
3. Vlaar APJ, Toy P, Fung M, et al. A consensus redefinition of transfusion-related acute lung injury. Transfusion. 2019;59(7):2465-76.
4. Wiersum-Osselton JC, Whitaker B, Grey S, et al. Revised international surveillance case definition of transfusion-associated circulatory overload: a classification agreement validation study. The Lancet Haematology. 2019;6(7):e350-e8.
5. Arvin AM, Fink K, Schmid MA, et al. A perspective on potential antibody-dependent enhancement of SARS-CoV-2. Nature. 2020;584(7821):353-63.
